# The effect of ferrous oral iron used in the treatment of iron deficiency on weight gain and appetite in adults: a prospective study

**DOI:** 10.1017/S1463423625100443

**Published:** 2025-09-26

**Authors:** Cansu Alici Yilmaz, Duygu Ayhan Baser, Hilal Aksoy, M. Merve Tengilimoglu-Metin

**Affiliations:** 1 Mamak Onur Uğurlu Family Health Center, Ankara, Turkey; 2 Department of Family Medicine, Hacettepe University Faculty of Medicine, Ankara, Turkey; 3 Department of Nutrition and Dietetics, Hacettepe University, Ankara, Turkey

**Keywords:** Appetite, iron deficiency, iron deficiency anaemia, oral iron therapy, primary health care, weight gain

## Abstract

**Aim:**

This study aimed to assess the impact of oral Fe^+2^ iron preparations on weight, body composition, metabolic, and appetite parameters in adults undergoing treatment for iron deficiency.

**Methods:**

In this observational prospective study, a total of 119 patients, aged 18–45, initiating Fe^+2^ iron therapy for iron deficiency within the last month at Family Medicine Outpatient Clinic, were included. Data on sociodemographic variables, health, dietary habits, anthropometric measurements, metabolic parameters, and appetite scores were collected. The Power of Food (PFS), Visual Analogue Scale (VAS), and Three-Factor Eating Questionnaire (TFEQ) were utilized for appetite assessment.

**Findings:**

After three months of iron treatment, a statistically significant increase was found in the mean values of Hb, Hct, MCV, ferritin, iron, and transferrin saturation; anthropometric measurements displayed a significant reduction in body weight, body mass index (BMI), fat percentage, waist circumference, hip circumference, and waist/hip circumference ratios post-treatment. Notably, VAS scores for certain food items decreased, while carbonated drinks VAS score increased. Appetite-related factors, as per PFS, exhibited a significant decrease in PFS factor 1 (food available), PFS factor 2 (food present).

**Conclusions:**

In conclusion, our findings indicate that oral Fe^+2^ iron preparations positively influence the treatment of iron deficiency anaemia by improving haematological parameters and concurrently leading to a significant reduction in body weight, BMI, and appetite scores related to specific food items. The study underscores the multifaceted impact of iron supplementation on both physiological and behavioural aspects, providing valuable insights for optimizing iron deficiency anaemia management.

## Introduction

Iron deficiency (ID), recognized by the World Health Organization (WHO) as a leading cause of anaemia, affects around 1.2 billion people globally (UNU, 2001). Iron deficiency anaemia (IDA) is prevalent, and non-anaemic ID is estimated to be twice as common (Camaschella, 2019, Kassebaum *et al.*, 2014). This condition, more frequent in women, not only poses individual health challenges but also constitutes a significant public health concern, diminishing quality of life and causing workforce loss (Erdem *et al.*, 2009). WHO’s 2019 anaemia prevalence study revealed anaemia rates of 29.9% in women, 36.5% in pregnant women, and 29.6% in non-pregnant women (WHO, 2022).

Treatment approaches should be individualized based on the underlying cause and ferritin levels. Oral iron supplementation is the standard treatment for ID, yet concerns about weight gain, attributed to increased appetite from iron preparations, persist in society (Galloway et al, 2002). This belief contributes to patient non-compliance, particularly concerning amid rising global obesity rates. The literature lacks a clear scientific answer regarding the relationship between iron replacement therapy and weight gain on adults. Existing studies on adults were limited, with a 2014 study on 21 women in Turkey showing decreased body mass index (BMI), weight, and waist circumference with improved lipid profiles in patients using oral iron preparations (Aktas *et al.*, 2014). Two studies on children produced conflicting results, with one from 1986 observing higher weight gain in children given iron (Aukett *et al.*, 1986) and another from 1994 in Indonesia concluding that iron supplements inhibited development (Idjradinata *et al.*, 1994). Ghrayeb and colleagues evaluated the effect of parenteral treatment on appetite, revealing decreased appetite and paradoxically increased Ghrelin hormone activity (Ghrayeb *et al.*, 2020). Despite various studies assessing appetite parameters and Ghrelin levels at different stages of ID, no study has evaluated the change in appetite hormones with oral iron supplementation. In this study, we aim to fill this gap by evaluating the effects of oral Fe^+2^ iron preparations on weight, BMI, body composition, metabolic parameters, and appetite parameters in adults.

## Material and methods

This observational prospective study was conducted in XXX University Faculty of Medicine, Family Medicine Outpatient Clinics between 25.01.2022 and 01.11.2022.

### Study population

The population of our study consisted of women aged 18–45 years who applied to XXX University Faculty of Medicine Family Medicine Outpatient Clinics. The monthly number of women aged 18–45 years who applied to the Family Medicine Outpatient Clinic was 455. The necessary sample size was calculated as a minimum of 92 individuals, with 95% power and 5% significance via G*Power 3.1.9.2 package. Inclusion criterion were;

- Women aged 18–45 years
- Serum ferritin value <15 μg/L
- Patients who were started Fe^+2^ oral iron preparation for ID within the last one month

### Ethical issues

The ethics committee approval of the study was obtained with the decision of Hacettepe University Clinical Research Ethics Committee dated 28.12.2021 and numbered 2021/30-10 (KA-21079).

The approval of the research by the Turkish Medicines and Medical Devices Agency was obtained on 25.01.2022.

The research is supported by the Scientific Research Projects Coordination Unit with the decision taken at the meeting dated 09.09.2022 and numbered 2022/15 (Project ID: 19916).

### Participants and recruitment

Overall, 212 women were reached during the study. However, some participants were excluded for specific reasons: eight due to a Beck Depression Scale score of 17 or higher, twelve for having a MET score above 3000 on the IPAQ, three due to chronic disease, and twelve for B12 deficiency. Additionally, 58 women were lost to follow-up. Therefore, 119 women aged 18–45 years who applied to Family Medicine Outpatient Clinics were included in the study. A list of all exclusion criteria are presented in Supplement 1.

### Procedure

In the study, a data form prepared to obtain exclusion criteria and descriptive data and some blood tests were applied to women who applied to the outpatient clinic, who were known to have started Fe^+2^ iron preparation (for its faster absorption) due to iron deficiency in the last 1 month and who agreed to participate in the study. At first evaluation (visit) anthropometric measurements were assessed via TANITA (after 3 hours later from waking up, before any food or drink consumption), metabolic parameters, appetite parameters (Ghrelin and Peptide YY levels) were measured, while sociodemographic characteristics, general health status, dietary habits, The Power of Food (PFS), Visual Analogue Scale (VAS), and Three-Factor Eating Questionnaire (TFEQ), Beck Depression Scale (BDS), and IPAQ were surveyed in the questionnaire.

At the three-month follow-up, measurements included TFEQ, PFS, VAS, anthropometrics, metabolic parameters and appetite parameters.

Response to oral iron therapy and changes in weight and appetite were analysed. The scales used in the case follow-up form are presented in Supplementary 1.

#### Ghrelin and peptide Y levels

Ghrelin and Peptide-YY plasma levels, which indicate appetite, were measured under hunger and satiety conditions before and after Fe^2+^ oral iron treatment.

For each subject, two blood samples (6 mL each) were collected in Ethylenediaminetetraacetic acid tubes. To prevent the degradation of acylated ghrelin and Peptide Y, 0.05 mL of 1N HCl and 0.01 mL of Phenylmethylsulfonyl fluoride per 1 mL of plasma were added to the samples. The samples were then centrifuged at 3500 RPM for 15 minutes at 4°C within one hour of collection. After centrifugation, the plasma was stored at −80°C for later analysis. Ghrelin levels were measured as total ghrelin using a commercial Ghrelin (Human) EIA Kit (Phoenix Pharmaceuticals, Inc., USA), and results were calculated using the standard graphic method.

#### Metabolic parameters

Complete blood count, iron, total iron binding capacity, iron saturation, transferrin, and ferritin levels were measured before and after Fe^2+^ oral iron treatment. Blood samples were analysed using an automated haematology analyser (Sysmex XN-1000) for complete blood count, while iron-related parameters were assessed using a chemiluminescence immunoassay with a Roche Cobas c501 analyser.

#### Anthropometric measurements

Anthropometric assessments, including weight, BMI, fat percentage, waist circumference, and hip circumference, were conducted before and after Fe^2+^ oral iron treatment.

Weight, BMI, and fat percentage were measured using the TANITA MC-780 analyser. Waist and hip circumferences were measured with a non-stretchable measuring tape. BMI was calculated as weight (kg) divided by height (m^2^).

### Data analysis

IBM SPSS v23.0 Statistics software version (IBM Corp., Armonk, NY, USA) was used to analyse all data. Shapiro-Wilk test was used to assess normality in the evaluation of the data. According to normality distributions, mean, median, standard deviation, minimum, maximum, interquartile distribution value for continuous variables and frequency table for categorical data were used. The scales used in the study were analysed to obtain scale scores. One-way analysis of variance, Friedman test and Cochran’s Q tests were used to compare the parameters between visits (0-3 month visits) according to the normality distributions of the values. A value of p < 0.05 was accepted as the level of significant.

## Results

Sociodemographic characteristics of the participants (*N* = 119) are summarized in Table 1. The mean age of the participants was 22.75 ± 4.734 years (min = 18, max = 44). It was determined that 45.4% (*n* = 54) of the participants received their first ID diagnosis less than 6 months ago.

**Table 1.** Sociodemographic characteristics, dietary habits, and general health status of the participants (*n* = 119)

		Number (*n*)			Percent (%)
**Marital Status**	Married	8			6.7
	Single	111			93.3
**Education Status**	Primary education	0			0
	Secondary education	0			0
	High school	101			84.9
	University	18			15.1
**Place of Residence**	City	96			80.7
	Town	20			16.8
	Village	3			2.5
**Profession/Job**	Have not a profession	0			0
	Student	101			84.9
	Have a profession	18			15.1
**Appetite Status** **(Self assesment)**	Good	75			63
	Medium	41			34.5
	Bad	3			2.5
**Additional Vitamin and Mineral Usage Status**	Regular Users	17			14.3
	Irregular Users	87			73.1
	None-users	15			12.6
**Probiotic, Nutritional Supplement Usage Status**	Yes	3			2.5
	No	116			97.5
**Time to First ID Diagnosis**	<6 months	54			45.4
	6 months-1 year	13			10.9
	1–3 years	16			13.4
	3–10 years	32			26.9
	>10 years	4			3.4
**Investigation for The Cause of ID**	Yes	5			4.2
	No	114			95.8
**Previous Iron Treatment**	Yes	69			58
	No	50			42
	**Mean**		**SD**	**Min**	**Max**
**Age**	22.75		4.734	18	44
**Daily Tea Consumption** **(cups/day)**	2.31		1.812	0	10
**Daily Coffee Consumption** **(cups/day)**	1.37		0.999	0	4
**Iron Treatment starting time (Months ago) (** ***n*** **= 69)**	21.29		19.840	1	120
**Duration of Iron Treatment (Months) (** ***n*** **= 69)**	2.75		1.744	1	12

**SD:** Standard Deviation; **Min**: Minimum; **Max:** Maximum; **ID**: Iron Deficiency.

Table 1. Sociodemographic characteristics, dietary habits, and general health status of the participants

Blood values and anthropometric measurement analysis table of the participants are given in Table 2. A statistically significant increase was found in the mean values of Hb, Hct, MCV, ferritin, iron, and transferrin saturation after iron (+2) treatment (*p* < 0.05). A statistically significant decrease was found in the mean values of red cell distribution width (RDW), total iron binding capacity, unsaturated iron binding capacity and transferrin values after iron (+2) treatment (*p* < 0.05).

**Table 2.** Comparison of blood values and anthropometric measurements before and after iron replacement therapy

	Before Iron Treatment				After Iron Treatment				
	Mean ± SD	Min-Max	Median	IQR_25–75_	Mean ± SD	Min-Max	Median	IQR_25–75_	*p*
**Hb**	12.99 ± 1.18	9.50–15.50	13.20	12.20-13.70	13.48 ± 0.98	9.30–15.60	13.50	12.90–14.10	**<0.001**
**Hct** ^*^	38.39 ± 3.06	30.20–45.50			39.36 ± 2.80	29.30–45.80			**0.001**
**MCV**	83.34 ± 5.62	67.20–92.20	84.40	79.30–86.80	85.73 ± 4.54	70.10–93.30	86.10	83.30–88.50	**<0.001**
**RDW**	14.43 ± 1.40	12.30–20.50	14.00	13.40–15.20	14.03 ± 1.15	12.10–18.30	13.80	13.30–14.50	**<0.001**
**Ferritin**	7.57 ± 3.39	1.80–14.80	6.70	5.20–10.20	22.37 ± 12.76	2.40–76.70	18.90	13.60–28.90	**<0.001**
**Iron**	70.53 ± 38.72	9.00–174.00	65.00	37.00–92.00	104.89 ± 57.83	25.00–349.00	91.00	65.00–128.00	**<0.001**
**TIBC**	412.75 ± 54.71	294.00–553.00	412.00	376.00–453.00	368.58 ± 47.88	258.00–514.00	362.00	337.00–393.00	**<0.001**
**UIBC**	339.43 ± 78.30	169.00-517.00	336.00	279.00–399.00	259.44 ± 81.18	21.00–472.00	261.00	211.00–312.00	**<0.001**
**Transferrin saturation**	18.26 ± 10.74	4.00–47.00	17.00	9.00–25.00	29.09 ± 16.04	5.00–94.00	25.00	19.00–35.00	**<0.001**
**Transferrin**	323.31 ± 50.49	232.00–469.00	321.00	285.00–352.00	291.36 ± 49.12	199.00–455.00	280.00	258.00–316.00	**<0.001**
**Weight**	58.77 ± 9.26	41.20–86.00	58.40	52.00–62.50	58.00 ± 9.07	40.10–84.90	57.70	51.40–62.80	**<0.001**
**BMI**	21.57 ± 2.98	16.50–29.70	21.10	19.50–23.30	21.36 ± 2.97	16.10–29.70	21.20	19.00–23.30	**<0.001**
**Fat percentage** ^*^	25.47 ± 6.13	8.70–39.10			24.45 ± 6.64	8.60–40.00			**<0.001**
**Waist circumference**	70.18 ± 6.92	58.00–88.00	69.00	64.00–75.00	69.55 ± 6.82	58.00–87.00	69.00	64.00–73.00	**<0.001**
**Hip circumference**	97.63 ± 8.19	74.00–118.00	98.00	92.00–102.00	97.27 ± 8.24	74.00–120.00	98	92.00–101.00	**0.001**
**Waist to hip ratio**	0.72 ± 0.05	0.58–0.93	0.70	0.68–0.76	0.72 ± 0.05	0.57–0.92	0.70	0.68–0.75	**<0.001**

**Hb:** Haemoglobin; **Hct**: Haematocrit; **MCV**: Mean Corpuscular Volume; **RDW:** Red cell distribution width; **TIBC**: Total Iron Binding Capacity;

**UIBC:** Unsaturated Iron Binding Capacity; **BMI:** Body Mass Index; **SD:** Standart Deviation; **Min:** Minimum; **Max**: Maximum; **IQR:** Interquartile Range;

* Parametric analyses were done.

The bold values indicate statistically significant *p*-values (*p* < 0.05).

When the results of the anthropometric measurements of the participants before and after iron treatment were compared, a statistically significant decrease was found in all parameters we examined after iron replacement therapy and is shown in Table 2 (Supplementary-2). A statistically significant decrease was found in the mean values of body weights, BMI, fat percentage, waist circumference, hip circumference, and waist/hip circumference ratios after iron (+2) treatment (*p* < 0.05).

Table 2. Comparison of blood values and anthropometric measurements before and after iron replacement therapy

The VAS data of the participants before and after iron replacement are shown in Table 3. Accordingly, VAS scores of chocolate and chocolate products, cream cakes and pastry products, chips, nuts decreased after iron replacement, and the difference was statistically significant (*p* < 0.05). Carbonated drinks VAS score increased after iron replacement and the difference was statistically significant (*p* < 0.05).

**Table 3.** Visual analogue scale comparison before and after iron replacement therapy

	Before Iron Treatment				After Iron Treatment				
Nutrients	Mean ± SS	Min-Max	Median	IQR_25–75_	Mean ± SD	Min-Max	Median	IQR_25–75_	*p*
**Chocolate and chocolate products**	7.02 ± 2.18	2–10	7	6–9	6.34 ± 2.19	1–10	7	5–8	**<0.001**
**Cream cakes and bakery products**	5.93 ± 2.42	0–10	6	4–8	5.43 ± 2.22	0–10	5	4–7	**0.005**
**Chips**	5.08 ± 2.84	0–10	5	3–7	4.83 ± 2.72	0–10	5	3–7	**0.025**
**Carbonated drinks**	3.06 ± 2.67	0–10	2	1–5	3.55 ± 2.68	0–10	3	1–6	**0.002**
**Fast-food**	5.45 ± 2.7	0–10	6	4–7	5.28 ± 2.29	0–10	5	3–7	0.287
**French fries**	5.83 ± 2.35	0–10	6	4–8	5.97 ± 2.40	0–10	6	4–8	0.755
**Bread types**	4.02 ± 2.44	0–10	4	2–5	4.24 ± 2.32	0–10	4	3–6	0.132
**Pasta**	5.09 ± 2.51	0–10	5	3–7	5.25 ± 2.53	0–10	5	3–7	0.161
**Pastries**	5.71 ± 2.15	1–10	6	4–7	5.51 ± 2.23	0–10	5	4–7	0.138
**Nuts**	5.61 ± 2.38	0–10	6	4–7	5.22 ± 2.30	0–10	5	3–7	**0.021**
**Core**	3.16 ± 2.73	0–10	3	1–5	3.34 ± 2.54	0–10	3	1–5	0.224
**Ice cream**	5.35 ± 2.79	0–10	5	3–7	5.65 ± 2.82	0–10	6	4–8	0.204
**Fruit**	6.41 ± 2.58	0–10	6	5–8	6.43 ± 2.46	0–10	6	5–8	0.851

**SD:** Standard Deviation; **Min:** Minimum; **Max**: Maximum; **IQR:** Interquartile Range.

The bold values indicate statistically significant *p*-values (*p* < 0.05).

Table 3. Visual Analog Scale comparison before and after iron replacement therapy

The PFS data of the participants before and after iron replacement are shown in Table 4. When the results of the participants’ PFS before and after iron therapy were compared, the median BDS score was found to be 3.00 (2.73–3.53) before iron therapy and 2.50 (2.00–3.17) after iron therapy and the decrease in PFS score was found to be statistically significant (*p* < 0.05). When the sub-factors of the PFS were analysed, a statistically significant decrease was found in the mean of PFS factor 1 (food available), PFS factor 2 (food present) (*p* < 0.05) (Supplementary-2).

**Table 4.** Comparison of Power of Food Scale and Three-Factor Eating Questionnaire before and after iron replacement therapy

	Before Iron Treatment				After Iron Treatment				
	Mean ± SD	Min-Max	Median	IQR_25–75_	Mean ± SD	Min-Max	Median	IQR_25–75_	*p*
**PFS mean score**	3.16 ± 0.65	1.53–5.00	3.00	2.73–3.53	3.02 ± 0.69	1.20–5.00	2.50	2.00–3.17	**<0.001**
**PFS factor 1 (food available)**	2.70 ± 0.87	1.17–5.00	2.67	2.00–3.33	2.58 ± 0.84	1.00–5.00	2.50	2.00–3.17	**0.006**
**PFS factor 2 (food present)**	3.39 ± 0.82	1.00–5.00	3.25	2.75–4.00	3.18 ± 0.87	1.00–5.00	3.00	2.75–3.75	**0.001**
**PFS factor 3 (food taste)**	3.51 ± 0.77	1.60–5.00	3.60	3.00–4.00	3.42 ± 0.77	1.60–5.00	3.60	3.00–4.00	0.056
TFEQ score*	49.72 ± 5.99	33.00–62.00			50.08 ± 5.83	36.00–62.00			0.303
**TFEQ factor 1**	12.92 ± 1.44	9.00–16.00	13.00	12.00–14.00	12.99 ± 1.46	9.00–17.00	13.00	12.00–14.00	0.673
**TFEQ factor 2**	7.73 ± 2.66	3.00–12.00	8.00	6.00–10.00	7.65 ± 2.75	3.00–12.00	8.00	6.00–9.00	0.658
**TFEQ factor 3**	17.23 ± 2.50	11.00–24.00	17.00	15.00–19.00	17.55 ± 2.24	12.00–23.00	17.00	16.00–19.00	0.103
**TFEQ factor 4**	11.85 ± 2.56	4.00–16.00	12.00	10.00–14.00	11.89 ± 2.34	5.00–16.00	12.00	11.00–14.00	0.666

**SD:** Standard Deviation; **Min:** Minimum; **Max**: Maximum; **IQR:** Interquartile Range; **PFS**: Power of Food Scale; **TFEQ:** Three-factor eating questionnaire

*Parametric analyses were done.

The bold values indicate statistically significant *p*-values (*p* < 0.05).

Table 4. Comparison of PFS and TFEQ before and after iron replacement therapy

When the results of the participants’ TFEQ before and after iron treatment were compared, no statistically significant difference was found in the total score and sub-factors of TFEQ (Table 4) (Supplementary-2).

When the results of the participants’ hunger and satiety Ghrelin and Peptide Y values before and after iron treatment were compared, the decrease in hunger Ghrelin level after iron replacement was found to be statistically significant (*p* < 0.05) (Table 5).

**Table 5.** Comparison of hunger and satiety Ghrelin and Peptide Y values before and after iron replacement therapy

	Before treatment		After treatment		*p*
	Median	IQR	Median	IQR	
Ghrelin-hunger	3,02	2,44	2,71	2,42	**0.009**
Ghrelin-satiety	3,05	2,69	2,81	2,63	0.158
Paptit Y- hunger	435,74	467,55	397,65	344,09	0.925
Peptit Y- satiety	380,01	406,145	409,60	415,84	0.363

*Wilcoxon Signed Ranks Test.*

The bold values indicate statistically significant *p*-values (*p* < 0.05).

Table 5. Comparison of hunger and satiety Ghrelin and Peptide YY values before and after iron replacement therapy

## Discussion

In our study assessing the impact of Fe+2 oral iron preparations on weight gain and appetite in adults, a comprehensive evaluation utilizing VAS, PFS, TFEQ, Ghrelin, and Peptide YY values revealed a significant decrease in most appetite parameters and anthropometric measures post-treatment. Assessing appetite with multiple scales and markers provides a more complete understanding of appetite changes, as each tool measures a different aspect of appetite.

In iron therapy, response variation is influenced by erythropoietin stimulation and absorption rates. The guidelines of Turkish Society of Hematology (2022) suggest a 1-2 g/dL increase in Hb values within 2–4 weeks of oral iron treatment for IDA, advocating a blood count 2–4 weeks post-initiation (Ünal *et al.*, 2011). In our 3-month study, the analysis revealed a statistically significant increase in haemoglobin (Hb), haematocrit (Hct), mean corpuscular volume (MCV), ferritin, iron, and transferrin saturation levels post-treatment. Conversely, a significant decrease was observed in RDW, total iron-binding capacity, unsaturated iron-binding capacity, and transferrin levels. These results are consistent with the expected physiological response to iron supplementation, where improved iron status leads to better haemoglobin synthesis and overall red blood cell health. However, it is crucial to note that these results were derived from univariate analyses, which do not account for potential confounding factors or interactions between variables. Future studies should incorporate multivariate analyses to provide a more comprehensive understanding of these relationships.

The statistical analysis of anthropometric measurements pre and post iron replacement therapy demonstrated significant decreases in body weight, BMI, fat percentage, waist circumference, hip circumference, and waist-to-hip ratio. While literature predominantly explores paediatric studies on iron and weight gain, our research on 119 adult participants contradicts the belief that oral iron preparations induce weight gain (Aukett *et al.*, 1986, Latham *et al.*, 1990, Soliman *et al.*, 2009, Angeles *et al.*, 1993). Notably, a study on women by G. Aktas et al. aligns with our findings, reporting decreased body weight, BMI, and waist circumference after iron replacement (Aktas *et al.*, 2014). Contrary to misconceptions, gastrointestinal side effects, revealed in a meta-analysis of 43 studies, including 20 placebo and 23 IV iron comparisons, did not lead to weight gain (Tolkien *et al.*, 2015). Symptoms such as constipation, nausea, and diarrhoea may impact dietary habits, contributing to decreased anthropometric measurements. Additionally, the taste disturbance from IDA and iron-induced free radicals causing a cellular response might influence reduced appetite and caloric intake (Knutson *et al.*, 2000). The findings of our study suggest that iron supplementation might influence body composition, possibly through improved metabolic efficiency or changes in energy expenditure. However, similar to the haematological markers, the clinical significance of these changes needs to be contextualized. For instance, a reduction in waist circumference and BMI could have positive implications for cardiovascular risk, yet the magnitude of these changes must be carefully interpreted.

In our study, we assessed the impact of iron treatment on appetite using the VAS score, PFS, and TFEQ. A comparison of pre- and post-iron replacement data revealed reduced cravings for chocolate, cream cakes, pastry products, and nuts. Interestingly, there was an increase in the desire for carbonated drinks, which may have been influenced by physicians advising patients on the enhanced absorption of iron in an acidic environment, leading to higher consumption of these beverages. No significant changes in VAS scores were observed for fast food items, French fries, bread varieties, pasta, pastries, seeds, ice cream, or fruit post-iron replacement.

Taste disturbances, often linked to iron deficiency (ID), are known to be reversible with iron replacement therapy (Miuchi *et al.*, 2008). Individuals with ID commonly experience a reduced ability to recognize tastes, particularly sweet, salty, sour, and umami (Jeon *et al.*, 2021). Our findings of reduced cravings for spicy and sweet foods after iron replacement may be due to improved taste sensation following ID correction, which allowed patients to be satisfied with smaller amounts of food. Additionally, patients experiencing gastrointestinal side effects may have avoided certain reflux-inducing foods, such as chocolate, while still consuming others like French fries and carbonated drinks (Surdea-Blaga *et al.*, 2019).

In our study, the mean PFS scores of the participants before and after iron treatment were above 2.5 and it was observed that the participants had hedonic hunger; however, the mean PFS scores decreased after iron treatment, that is, their hedonic hunger decreased. In other words, the desire to consume discretionary foods without the need for energy decreased after iron treatment. When the sub-factors of the PFS were analysed, it was observed that the sub-scores of food availability and food availability decreased; the score of tasting foods did not change. In the literature, no study evaluating the relationship of hedonic hunger with anaemia, ID or iron therapy was found.

In our study, post-iron treatment, participants showed increased total scores on the TFEQ scale, indicating a controlled restriction of eating behaviours. While studies examining TFEQ in relation to anaemia, iron deficiency (ID), or iron treatment are limited, our VAS results revealed a decrease in interest for various nutrients following iron supplementation. This reduced appetite, observed in eating behaviours, was coupled with a diminished desire for hedonic foods, those consumed for pleasure rather than nutritional value. Previous research has established a connection between hedonic hunger and the regulation of eating behaviours (Ergülen *et al.*, 2001; Schultes *et al.*, 2010; Thomas *et al.*, 2013; Ribeiro *et al.*, 2018). However, our study found no significant changes in emotional eating, uncontrolled eating, cognitive restraint, or hunger sensitivity post-iron treatment.

Our study indicates a decrease in appetite-related parameters following iron treatment, with a notable reduction in Hunger-Ghrelin levels. The observed inverse relationship between iron supplementation and appetite suggests a potential regulatory role of ghrelin, an appetite-stimulating hormone. Future investigations could explore the specific pathways through which iron influences ghrelin secretion, considering the multifaceted nature of appetite regulation and potential interactions with other hormones or signalling pathways. Longitudinal studies, such as one published in 2020 with 56 IDA patients, reported increased Short Nutritional Assessment Questionnaire scores and ghrelin levels after ID treatment, suggesting enhanced appetite (Ghrayeb *et al.*, 2020). However, our study’s appetite questionnaire results differ, highlighting the need for further research to reconcile these discrepancies.

The strengths of this study, when the studies in the literature were examined, studies evaluating the effect of parenteral iron therapy on appetite in adult patients and the effect of oral iron therapy on appetite in paediatric patients were found, but only one study with a limited number of studies on the effect of oral iron preparations on appetite in adults was found. Our study contributes to the literature in terms of sample size. There is no study that examined patients holistically with appetite questionnaires and appetite metabolites (ghrelin, peptide YY). Our study will provide evidence in these aspects.

Our study has limitations, including the absence of a scale to assess medication use and patient regularity. Another limitation is the exclusion of male patients without chronic diseases or underlying causes, reflecting a gap in reaching a broader demographic. Although we achieved the target sample size, the study’s focus on a university hospital resulted in a higher student population, limiting the generalizability of our findings to a single city in Türkiye. Additionally, the 3-month follow-up period poses a constraint. Another important limitation of our study was to the lack of multivariate analysis and the difficulty in accounting for confounders. Future research, considering these limitations, should confirm our findings across different cultures and expanded samples.

## Conclusion

This study demonstrated a significant association between Fe^+2^ oral iron preparations and a marked reduction in appetite, accompanied by favourable decreases in body weight, BMI, fat percentages, waist circumference, hip circumference, and waist/hip ratios. Future investigations should delve into the underlying mechanisms of these changes in appetite and body composition post- Fe^+2^ oral iron supplementation. Exploring long-term effects and variations across demographics could enhance our understanding. Further research on optimal dosage, duration, and potential interactions with other factors influencing appetite and weight is recommended.

## Supporting information

Alici Yilmaz et al. supplementary material 1 Alici Yilmaz et al. supplementary material

Alici Yilmaz et al. supplementary material 2 Alici Yilmaz et al. supplementary material

## Supplementary material

The supplementary material for this article can be found at https://doi.org/10.1017/S1463423625100443
