# Supplementary material for: The effect of ferrous oral iron used in the treatment of iron deficiency on weight gain and appetite in adults: a prospective study
Source: Prim Health Care Res Dev. 2025 Sep 26;26:e82. doi: 10.1017/S1463423625100443 (PMC12555074; doi:10.1017/S1463423625100443)
Supplement: Alici Yilmaz et al. supplementary material 1 — Alici Yilmaz et al. supplementary material [file S1463423625100443sup001.docx]

**FIGURES**

**Figure 1**. Flow Chart

**Figure 2.** Comparison of anthropometric measurements before and after iron treatment (Mean values)

**Figure 3.** Comparison of PFS scores before and after iron treatment (Mean values)

**Figure 4.** Comparison of TFEQ scores before and after iron treatment (Mean values)
