# Supplementary material for: The effect of ferrous oral iron used in the treatment of iron deficiency on weight gain and appetite in adults: a prospective study
Source: Prim Health Care Res Dev. 2025 Sep 26;26:e82. doi: 10.1017/S1463423625100443 (PMC12555074; doi:10.1017/S1463423625100443)
Supplement: Alici Yilmaz et al. supplementary material 2 — Alici Yilmaz et al. supplementary material [file S1463423625100443sup002.docx]

***Inclusion criteria:***

- Women aged 18-45 years
- Serum ferritin value <15 μg/L
- Patients who were started Fe+2 oral iron preparation for ID within the last one month

***Exclusion criteria:***

- Those who started using iron preparations before the last one month*
- Malignancies*
- Gastrointestinal Diseases*
- Hypothyroidism**
- Vitamin B12 deficiency**
- Pregnancy*
- Obesity (BMI>30)
- Anorexia
- Polycystic ovary syndrome*
- Diabetes Mellitus**
- Those who have undergone bariatric operation*
- Chronic disease anemia**
- Pernicious anemias**
- Steroid and androgenic steroid users*
- Vitamin complex users*
- Hereditary hemoglobinopathies*
- Non-reference results in laboratory test results**

* Patient declaration will be taken as basis

** To be checked at the first visit

***Beck Depression Inventory (BDI)***

The Turkish validity and reliability study of the Beck Depression Inventory developed by Beck and colleagues [13] to determine the risk for depression and to determine the level of depressive symptoms was conducted by Hisli [14]. It measures physical, emotional, cognitive, and motivational symptoms of depression. The aim of the scale is not to diagnose depression, but to objectively determine the degree of depression symptoms. A high total score indicates a high level or severity of depression. Those who scored 17 points and above were excluded.

***International Physical Activity Questionnaire*** *(IPAQ)*

The International Physical Activity Questionnaire (IPAQ) was developed by Craig et al. [11] to determine the physical activity levels and the validity and reliability study of the IPAQ in Turkey was conducted by Ozturk [12]. According to the total physical activity score, the physical activity levels of the women were categorized as “low, moderate and high”. Physical Activity Levels:

1. Low level: less than 600 MET-min/week.

2. Moderate level: between 600-3000 MET-min/week.

3. High level: above 3000 MET-min/week.

In our study, the IPAQ with high levels of activity was used as an exclusion criterion.

***Three-Factor Eating Questionnaire (TFEQ)***

The TFEQ is used to measure the levels of individuals’ conscious restraint actions towards eating, the levels of uncontrolled eating, and the degree of change in eating states according to mood. The TFEQ is 18-items self-report questionnaire, designed to evaluate four dimensions of eating behaviors as “cognitive restraint of eating”, “emotional eating”, “uncontrolled eating” and “perceived hunger”. A higher total sub-dimension score indicates a tendency for the eating behavior sub-dimension. The questionnaire was developed by Stunkard and Messick et al. (Stunkard & Messick, 1985) and tested for its Turkish validity and reliability (Kıraç et al., 2015). 15-16

A high mean score indicates controlled restriction in eating behavior. The higher the score for each psychological eating behavior, the stronger the individuals' degree of cognitive restraint, emotional eating, uncontrolled eating, and hunger sensitivity.

***Visual Analog Scale***

Ten centimeters continuous line VAS was used to determine subjective ratings for desire to eat some foods such as chocolate and cakes. Participants were instructed to place a vertical line to indicate their ratings and score different foods with VAS for the last one month to determine which food types they are more interested in. Maximum score was 10 points for VAS. The VAS scale was first developed by Freyd in 1923 [17].

***Power of Food (PFS)***

The "Power of Food Scale-PFS" was developed by Cappelleri et al [18] and the Turkish validity and reliability of the scale was conducted by Hayzaran M. et al. in 2018 [19]. It has three sub-factors that measure reactions to food situations:

1) Food availability; it is assumed that delicious food is available in the environment. Therefore, these items are the most abstract. This is because these items describe responses to a food environment in which food is always imaginatively present but not physically present.

2) Food present describes a situation in which palatable food is physically present in the environment but has not yet been tasted.

3) Food tasted describes the situation where palatable foods have only been tasted but not yet consumed in their entirety.

If the total score of the PFS is 2.5 and above, it is defined as hedonic hunger, and higher total scores indicate more hedonic hunger level. High scores indicate that the individual is more sensitive to the food environment and is psychologically controlled by food.
